# Meeting Sexual Partners Through Internet Sites and Smartphone Apps in Australia: National Representative Study

**DOI:** 10.2196/10683

**Published:** 2018-12-18

**Authors:** Lucy Watchirs Smith, Rebecca Guy, Louisa Degenhardt, Anna Yeung, Chris Rissel, Juliet Richters, Bette Liu

**Affiliations:** 1 Kirby Institute University of New South Wales, Sydney Sydney Australia; 2 National Drug and Alcohol Research Centre University of New South Wales, Sydney Sydney Australia; 3 Centre for Urban Health Solutions, Li Ka Shing Knowledge Institute St. Michael’s Hospital Toronto, ON Canada; 4 Sydney School of Public Health University of Sydney Sydney Australia; 5 School of Public Health and Community Medicine University of New South Wales, Sydney Sydney Australia

**Keywords:** dating websites, internet, mobile phone, sexually transmitted diseases, health risk behaviors

## Abstract

**Background:**

Studies have reported on the proportion of the population looking for potential sexual partners using internet sites and smartphone apps, but few have investigated those who have sex with these partners, arguably a more important target group for health promotion.

**Objective:**

This study aimed to determine the proportion of people who have had sex with someone they met on an internet site or a smartphone app in the previous year.

**Methods:**

We analyzed data from the 2012-2013 Second Australian Study of Health and Relationships, a nationally representative telephone survey of Australian residents aged 16-69 years (N=20,091). The participation rate for the telephone survey was 66.22%. The prevalence of looking for a potential partner, physically meeting, and having sex with someone first met through an internet site or a smartphone app was estimated. Multivariate logistic regression was used for men and women separately to determine demographic and behavioral factors associated with having had sex with someone met on an internet site or a smartphone app in the last year.

**Results:**

Overall, 12.09% of respondents had looked for potential partners using these technologies and 5.40% had done so in the last year. In the last year, 2.98% had met someone in person and 1.95% reported having had sex with someone first met on an internet site or a smartphone app. The prevalence of all behaviors was greater in men than in women and in younger respondents than in older respondents. Among sexually active men, factors associated with having had sex with someone met using internet sites or smartphone apps included identifying as gay or bisexual (adjusted odds ratio, AOR: 15.37, 95% CI 8.34-28.35), having either 2-3 or >3 sexual partners in the last year (AOR: 9.20, 95% CI 9.20-34.68 and AOR: 35.77, 95% CI 18.04-70.94, respectively), having had a sexually transmissible infection (STI) test in the past year (AOR: 2.02, 95% CI 1.21-3.38), or an STI in the last year (AOR: 3.15, 95% CI 1.25-7.97). Among sexually active women, factors associated with having had sex with someone met on an internet site or a smartphone app were as follows: having either 2-3 or >3 sexual partners in the last year (AOR: 32.01, 95% CI 13.17-77.78 and AOR: 71:03, 95 % CI 27.48-183.57, respectively), very low and low income (vs very high AOR: 3.40, 95% CI 1.12-10.35), and identifying as lesbian or bisexual (AOR: 2.27, 95% CI 1.04-4.49).

**Conclusions:**

More than a third of adults who had looked for potential partners using websites and apps each year had sex with such partners, and those who had done so were more sexually active, suggesting that dating and hookup websites and applications are suitable settings for targeted sexual health interventions.

## Introduction

A range of dating websites, accessible via the internet or smartphone apps, are now available to search for potential sexual partners. These sites first surfaced in 1995 with match.com and eHarmony in 2000; in 2009, Grindr was launched, targeting men who have sex with men, followed by Tinder in late 2012 (targeted more toward heterosexually active adults). Since then, increasing numbers of these apps have become available. People have various motivations for using these sites and apps; some may be searching for a life partner and others for just a one-off encounter. The platforms enable selection of partners based on preferred personal characteristics, and some sites use geospatial technology to allow the user to determine the geographical proximity of a potential partner (eg, both Grindr and Tinder are location-based hookup apps). Sites are also available for particular cultural groups, and some focus on certain sexual preferences. The sites are generally open to people aged ≥18 years.

Beyond that, dating sites have the potential to provide sexual health promotion interventions. However, there is little available information on how many people access these sites and what their characteristics are. To date, most studies of meeting partners online have recruited specific populations and used convenience-sampling strategies, such as targeting online users, gay venues, or health care settings [1-3]. However, these settings are not representative and may result in an overestimation of prevalence.

Furthermore, earlier studies reported on the proportion and characteristics of people who looked for partners using Web-based technologies (but may not have intended to have sex with them or actually have done so). However, the characteristics of people who have sex with these partners are of the greatest relevance for health promotion. Of the two population-level studies conducted to date, a study among Norwegian young people (aged 15-20 years) in 2009 found that 30% reported ever having had sex with someone they met online (but did not ask about the last year), and a British survey of adults (aged 16-74 years), conducted from 2010 to 2012, focused only on respondents looking for sexual partners in the last year, not whether respondents had sex with them [4,5].

In 2012-2013, the Second Australian Study of Health and Relationships (ASHR2) survey was conducted just after the introduction of Tinder and other geosocial dating apps [6]. ASHR2 is a national representative survey of the Australian population covering demographics, knowledge, attitudes, behaviors, and experiences related to sexual health. A series of questions about looking for, physically meeting, and having sex with people met on websites or smartphone apps were also asked [7,8], providing an opportunity to determine both the prevalence of Australian adults who had looked for potential partners on websites and apps and the characteristics of people who had sex with these partners in the past year.

## Methods

### Study Population

This study was a cross-sectional analysis of data from ASHR2. The methods of ASHR2 have been described elsewhere [7]. In brief, ASHR2 is a national survey of 20,091 Australian residents aged 16-69 years. Data were collected in 2012 and 2013 via a computer-assisted telephone survey by trained interviewers. The study sample was selected using a modified random digit dialing sampling frame, which combined random digit dialing of landlines with that of cell phones. The overall participation rate among eligible people was 66.22%; the study population has been shown to be broadly representative of the Australian population, except for an overrepresentation of people with postgraduate degrees [7].

To allocate resources efficiently and gather more information from those with potentially higher HIV and sexually transmissible infection (STI) risk, we administered interviews in two forms [7]. All respondents who reported no sexual partners or >1 sexual partner in the previous year or any lifetime same-sex experience were given a long-form interview, as were a 20.00% random sample of survey respondents who had reported having 1 partner in the previous year and no same same-sex experience; the remaining 80.00% of one-partner respondents were given the short-form interview. Questions asked only in the long-form interview included those on meeting and having sex with a partner met on websites and smartphone apps.

### Statistical Analysis

The estimates of prevalence included sexually active and sexually inactive respondents. However, the predictor analysis of factors associated with meeting and having had sex with someone met on websites or smartphone apps was restricted to sexually active respondents because many sexual health outcomes were queried only of sexually active survey respondents. For this study, people were considered sexually active if they had ≥1 partners (for vaginal or anal intercourse or oral or manual sex) of the same or other sex in the previous 12 months. Respondents who reported no lifetime sexual experience were coded as not sexually active.

**Questions related to use of internet and smartphone apps from the Second Australian Study of Health and Relationships.**

1. Have you ever used an internet site or smartphone application to look for potential partners? Have you done so in the past year?
2. In the last year have you met someone in person that you first met on an internet site?
3. And did you have sex with that person?

### Outcome Measure

The primary outcome of this study was having sex with someone met on a website or a smartphone app in the past year. We also calculated the proportion of people looking for partners and meeting someone in person who they first met on a website or mobile app. These outcomes were ascertained using the following questions collected in the long-form questionnaire. The questions’ exact wording is shown in Textbox 1.

The proportion of people searching for, meeting, and having sex with partners on websites and smartphone apps were calculated separately using descriptive statistics. Data were weighted according to the Australian population and the probability of being selected for the long-form questionnaire. The characteristics of respondents who reported searching for partners using these technologies were compared with respondents who reported having sex with someone met on a website or a smartphone app. A chi-square test was used to compare differences in distributions between groups for a range of covariates.

Univariate and multivariate logistic regression, weighted in accordance with study procedures, were used to examine factors associated with having had sex with someone met on a website or a smartphone app in the last year. All data were analyzed using Stata statistical software version 14. Variables significant at the *P*<.1 level in the univariate analysis were included in multivariate logistic regression analyses. Backward elimination of variables was then used to determine the final adjusted model.

The demographic covariates included in the models were age group (16-29 years, and then 10-year age groups up to 69 years), language spoken at home (English or other), annual household income: very low or low (<Aus $52,000), middle (Aus $52,001- Aus $83,000), high (Aus $83,001-Aus $125,000), and very high (>Aus $125,000), and area of residence (urban or rural and remote) according to the Accessibility/Remoteness Index of Australia [9].

The behavioral covariates included in models were levels of alcohol consumption (high or not, with high alcohol consumption classified as >28 standard drinks per week for men and >14 standard drinks per week for women), injecting drug use in the last year (yes or no), smoking status (never and former, or current), sexual identity (heterosexual or gay, lesbian, bisexual, and other), condom use at last event (used or did not use condoms), STI history the last year (no STI test, STI test, or STI diagnosis), and sexual partner numbers in the previous year (1, 2-3, or >3). The numbers of sexual partners included both male and female partners. In relation to STIs, respondents were asked whether they had had an STI in the past year and whether they had an STI test; these two questions were combined to provide the composite variable. The STIs included were pubic lice, genital warts, chlamydia, genital herpes, gonorrhea, and syphilis; in addition, for women, warts virus on Pap smear, pelvic inflammatory disease, bacterial vaginosis or gardnerella, and trichomoniasis were included and for men, nonspecific urethritis and anal warts were included [10].

### Ethical Approval

The study received La Trobe University’s (HEC 11-040) ethical approval, which was ratified by the ethics committees of the University of New South Wales, the University of Sydney, and the University of Sussex.

## Results

### Prevalence of Looking for Potential Partners on Websites and Smartphone Apps and Meeting Them in Person

Overall, 12.09% (2346/19,398) of respondents reported ever searching for potential partners on websites and smartphone apps (13.52% men [1320/9761], 10.65% women [1026/9637]) and 5.40% (1048/19,398) of respondents (7.01% men [685/9637] and 3.77% women [364/9636]) reported doing so in the last year. Table 1 shows that searching for potential partners using smartphone apps and websites in the last year was most common among people aged 16-29 years (8.42%, 435/5169) and decreased with increasing age to 1.87% (52/2785) among people aged 60-69 years. Furthermore, 4.92% (815/16586) of sexually active respondents used websites and smartphone apps to look for potential partners in the last year, and 8.28% (233/2811) of sexually inactive respondents did. The activity was more common among gay, lesbian, and bisexual respondents (25.32%, 172/680) than among heterosexual respondents (4.68%, 876/19715).

**Table 1:** Prevalence of looking for potential partners on websites and smartphone apps and prevalence of having sex with these partners.

Characteristics		Searched for potential partner (ever), %^a^ (95% CI)	Searched for potential partner (last year), %^a^ (95% CI)	Met in person (last year), %^a^ (95% CI)	Had sex (last year), %^a^ (95% CI)
All participants^b,c^		12.09 (11.24-13.00)	5.40 (4.89-5.97)	2.98 (2.63-3.37)	1.95 (1.69-2.25)
**Sex**					
	Men	13.52 (12.26-14.88)	7.01 (6.20-7.93)	3.70 (3.14-4.36)	2.54 (2.14-3.02)
	Women	10.65 (9.54-11.87)	3.77 (3.17-4.48)	2.19 (1.81-2.65)	1.35 (1.04-1.74)
**Sexually active in last year**					
	Yes	11.41 (10.46-12.44)	4.92 (4.35-5.55)	2.97 (2.58-3.42)	N/A^d^
	No	16.10 (14.58-17.74)	8.28 (7.16-9.56)	3.01 (2.36-3.85)	N/A
**Age group (years)**					
	16-29	14.24 (12.25-16.50)	8.42 (7.05-10.02)	4.73 (3.85-5.79)	3.03 (2.41-3.81)
	30-39	15.89 (13.71-18.35)	6.30 (5.07-7.80)	3.57 (2.68-4.73)	2.25 (1.66-3.05)
	40-49	11.83 (10.21-13.67)	4.78 (3.92-5.82)	2.40 (1.87-3.08)	1.79 (1.33-2.42)
	50-59	10.0 (8.54-11.67)	3.52 (2.78-4.45)	2.00 (1.42-2.80)	1.23 (0.79-1.92)
	60-69	5.84 (4.74-7.17)	1.87 (1.43-2.44)	0.98 (0.66-1.47)	0.54 (0.29-0.99)
**Sexual identity**					
	Heterosexual	10.98 (10.12-11.90)	4.68 (4.17-5.25)	2.40 (2.07-2.78)	1.45 (1.21-1.73)
	Homosexual or lesbian or bisexual	42.80 (37.97.0-47.78)	25.32 (21.40-29.69)	18.9 (15.48-22.87)	15.8 (12.66-19.55)
**Language spoken at home**					
	English only	12.14 (11.27-3.06)	5.26 (4.76-5.81)	2.98 (2.63-3.37)	2.02 (1.75-2.29)
	Other	11.44 (7.95-16.19)	7.47 (4.66-11.76)	2.94 (1.37-6.17)	0.89 (0.380-2.10)
**Annual household income**					
	Very low or low	11.89 (10.08-13.98)	6.60 (5.21-8.32)	3.98 (2.43-3.19)	2.49 (1.77-3.46)
	Middle	10.48 (8.57-12.75)	4.65 (3.62-5.96)	1.92 (1.39-2.64)	1.12 (0.75-1.73)
	High	9.52 (7.72-11.68)	2.43 (1.65-3.55)	0.94 (0.62-1.40)	0.55 (0.33-0.93)
	Very high	9.76 (7.98-11.)	2.70 (1.94-3.75)	1.58 (1.04-2.41)	1.03 (0.72-1.46)
**Area of residence^e^**					
	Urban	12.92 (11.82-14.09)	5.62 (4.97-6.36)	3.37 (2.90-3.91)	2.11 (1.78-2.51)
	Regional or remote	10.25 (8.98-11.7)	4.94 (4.14-5.88)	2.13 (1.71-2.64)	1.52 (1.17-1.96)
**High alcohol consumption**					
	No	12.47 (11.45-13.56)	5.51 (4.89-6.21)	2.91 (2.51-3.36)	1.86 (1.57-2.20)
	Yes	11.20 (9.72-12.87)	5.14 (4.27-6.18)	3.15 (2.28-3.99)	2.10 (1.61-2.75)
**Injected drugs in last year**					
	No	11.93 (11.07-12.85)	5.28(4.76-5.86)	2.93 (2.57-3.33)	191 (1.65-2.22)
	Yes	19.08 (14.21-25.13)	10.50 (7.43-14.63)	4.90 (3.00-7.90)	3.6 (2.00-6.43)
**Smoking status**					
	Never smoked/former	11.09 (10.15-12.10)	4.65 (4.10-5.27)	2.6 (2.25-3.01)	1.79 (1.51- 2.12)
	Current smoker	16.75 (14.63-19.12)	9.07 (7.63-10.74)	4.86 (3.79-6.21)	2.88 (2.20-3.77)
**STI^f^ testing in last year**					
	No test	9.13 (8.20-10.14)	3.32 (2.81-3.92)	1.70 (1.38-2.09)	1.18 (0.94-1.48)
	STI test	23.86 (20.38-27.73)	13.57 (11.20-16.35)	9.55 (7.73-11.76)	7.24 (5.84-8.93)
	STI diagnosis	36.96 (26.46-17.39)	26.00 (18.05-35.92)	21.13 (14.21-30.23)	16.88 (10.89-25.25)
**Condom use with most recent partner**					
	Used condoms	14.69 (12.50-17.18)	9.35 (7.79-11.17)	5.78 (4.69-7.13)	4.52 (3.55-5.74)
	Did not use	12.11 (10.49-13.94)	4.51 (3.66-5.54)	2.70 (2.12-3.45)	1.92 (1.51-2.44)
**Number of sexual partners in last year**					
	1	9.66 (8.80-10.61)	3.0 (2.6-3.6)	1.20 (0.93-1.54)	0.47 (0.31-0.69)
	2-3	33.99 (30.57-37.51)	25.4 (22.3-28.7)	16.9 (14.31-19.85)	12.40 (10.19-15.00)
	>3	39.44 (34.53-44.58)	33.0 (28.4-38.0)	27.32 (22.99-32.12)	23.91 (19.87-28.49)

^a^All proportions have been weighted to match the Australian population.

^b^N=19,398 (8184), weighted (unweighted) denominators

^c^Individuals with missing data are not shown; this was <5% for all variables except for income, which was incomplete for 24.5% of participants.

^d^N/A: not applicable.

^e^Accessibility/Remoteness Index of Australia.

^f^STI: sexually transmissible infection.

Having met in person was reported by 2.98% (578/19,398) of survey respondents (3.70% men [363/9761], 2.19% women [214/9637]), whereas having had sex with someone first met on a website or a smartphone app was reported by 1.95% (378/19,398) of respondents (2.54% men [248/9761], 1.35% women [130/9637]). Having had sex with someone met on an internet site or a smartphone app in the last year was the highest among respondents who identified as gay, lesbian, or bisexual (15.80%, 107/680) and also more frequent among people aged 16-29 years and 30-39 years, as well as those who had had an STI test or STI diagnosis in the past year, those with a higher number of sexual partners, and those who used a condom at their last sexual event (Table 1). Results from Table 1 stratified by sex are presented in Multimedia Appendices 1 and 2.

When restricted to sexually active respondents,11.41% (1893/16586) had ever searched for potential partners online, 4.92% (815/16586) had done so in the last year, and 2.97% (493/16586) had met with someone in person in the last year (Table 1).

Table 2 displays the proportion of respondents who had sex with someone met on a website or a smartphone app, among those who reported using websites and smartphone apps to search for potential partners in the last year overall and according to selected characteristics. Overall, slightly over one-third (36.07%, 378/1048) of those who searched for partners also reported having had sex with someone they met using these technologies in the last year. There were no statistical differences by sex. Differences were observed for sexual identity, with gay, lesbian, and bisexual respondents being more likely also to report having sex with someone they met on a website or a smartphone app, than heterosexual respondents (62.41% [107/172] vs 30.89% [271/876], *P*<.001). Differences were also seen for STI testing history (no test: 35.89% [170/473], STI test: 53.77% [171/318], and STI diagnosis: 64.94% [33/51], *P*<.001), condom use at most recent event (used condoms: 48.43%[151/312] vs not used: 42.85% [122/285], *P*<.001), and numbers of sexual partners (1 partner: 15.36%, [83/541], 2-3 partners: 48.82% [150/307], and >3 partners 72.44% [145/200], *P*<.001).

**Table 2:** Proportion of respondents reporting having had sex with someone met on a website or a smartphone app among people who searched for partners on websites and smartphone apps.

Characteristics		%^a^ (95% CI)	*P* value
All participants^b^		36.07 (31.64-40.75)	N/A^c^
**Sex**			
	Men	36.28 (30.90-42.02)	.90
	Women	35.68 (28.20-43.93)	N/A
**Sexual identity**			
	Heterosexual	30.89 (26.19-36.03)	<.001
	Homosexual or lesbian or bisexual	62.41 (53.24-70.75)	N/A
**STI^d^ testing in the last year**			
	No test	35.89 (28.86-43.59)	<.001
	STI test	53.77 (43.94-63.31)	N/A
	STI diagnosis	64.94 (46.76-79.61)	N/A
**Condom use with most recent partner**			
	Used condoms	48.43 (39.7 57.2)	<.001
	Did not use	42.85 (33.5-52.8)	N/A
	Missing or don’t know or refused	23.26 (18.0-29.5)	N/A
**Number of sexual partners in the last year**			
	1	15.36 (10.50-21.94)	<.001
	2-3	48.82 (41.66-56.02)	N/A
	>3	72.44 (63.95-79.57)	N/A

^a^All data have been weighted to match the Australian population.

^b^n=1048 (720) weighted (unweighted) denominators.

^c^N/A: not applicable.

^d^STI: sexually transmissible infection.

Among the respondents who had had sex with someone they met on a website or a smartphone app, 62.88% (363/578) were male and 37.12% (214/578) were female. The majority of people who reported having had sex with someone met on a website or a smartphone app identified as heterosexual (77.75%, 129/578). In terms of STI testing, 41.91% (242/578) reported not having had an STI test in the previous year, 38.81% (224/578) reported having had an STI test, and 7.24% (42/578) reported being diagnosed with an STI in the last year (12.03%, 70/578) of respondents either refused to answer, could not recall, or were not asked). Regarding the numbers of sexual partners, 36.45% (211/578) had 1 sexual partner in the last year, 35.28% (204/578) had 2 or 3 sexual partners, and 28.27% (163/578) had >3 sexual partners.

### Correlates of Having Sex With Someone Met Online

#### Men

Among sexually active males, most respondents were heterosexual (97.34%, 8339/8567), spoke English at home (93.80% (8012/8526), and resided in an urban area (68.04%, 5788/8526). Age was distributed as follows: 24.24% (2077/8567) were aged 16-29 years, 21.03% (1802/8567) were aged 30-39 years, 22.12% (1896/8567) were aged 40-49 years, 18.66% (1599/8567) were aged 50-59 years, and 13.92% (1192/8567) were aged 60-69 years. Most respondents had either very high (30.61%, 2623/8567) or high (20.06%, 1719/8567) annual household incomes.

The peak reporting of having had sex with someone met on a website or a smartphone app was among sexually active men aged 16-29 years (4.78%, 100/2077). This declined with increasing age to 0.81% (10/1192) among sexually active men aged 60-69 years. Reporting having had sex with someone met on a website or a smartphone app was substantially higher among homosexual and bisexual men than among heterosexual men (36.23% [83/228] vs 1.86% [155/8339]). In terms of sexual practices, 1.43%(106/7468) of sexually active men with no STI test in the last year reported having had sex with someone met on a website or a smartphone app, compared with 10.92% (111/1017) of men with an STI test in the last year and 31.09% (20/66) of men who reported having had an STI diagnosis in the last year. In addition, 0.61% (45/7352) of sexually active men with 1 sexual partner in the last year reported having had sex with someone met on a website or a smartphone app, compared with 11.85% (91/772) of those with 2-3 sexual partners and 22.85% (101/444) of those with >3 sexual partners in the last year.

**Table 3:** Sociodemographic and other behavioral characteristics of having sex with someone met on a website or a smartphone app among sexually active men.

Characteristics^a,b^		%^c^ (95% CI) in subgroup	%^c^ (95% CI) outcome	Odds ratio (95% CI)	*P* value	Adjusted odds ratio (95% CI)^d^	*P* value
**Age group (years)**							
	16-29	24.24 (22.01-26.61)	4.78 (3.54-6.45)	1.60 (0.95-2.68)	.08	0.43 (0.23-0.82)	.01
	30-39	21.03 (19.05-23.2)	3.05 (2.06-4.55)	Referent		Referent	
	40-49	22.12 (20.26-24.1)	2.39 (1.68-3.41)	0.78 (0.45 - 1.34)	.36	1.01 (0.51-2.01)	.97
	50-59	18.66 (17.03-20.41)	1.76 (1.13-2.75)	0.57 (0.31-1.05)	.07	0.62 (0.28-1.40)	.25
	60-69	13.96 (12.59-15.46)	0.81(0.46-1.43)	0.26 (0.13-0.52)	<.001	0.28 (0.12-0.65)	.003
**Sexual identity**							
	Heterosexual	97.34 (96.86-97.75)	1.86 (1.48-2.35)	Referent		Referent	
	Homosexual, bisexual or other	2.66 (2.25-3.14)	36.23 (28.83-44.34)	30.01 (19.88-45.31)	<.001	15.37 (8.34-28.35)	<.001
**Language spoken at home**							
	English only	93.80 (92.41-94.95)	2.87 (2.44-3.50)	N/A^e^	N/A	N/A	N/A
	Other	6.20 (5.05-7.59)	—^f^	N/A	N/A	N/A	N/A
**Annual household income**							
	Very high	30.65 (28.46- 32.92)	30.61 (28.43-32.89)	Referent	N/A	N/A	N/A
	High	20.06 (18.20 -22.05)	0.67 (0.34-1.32)	0.49 (0.22-10.9)	.08	N/A	N/A
	Middle	16.19 (14.45-18.10)	1.79 (1.11-2.89)	1.28 (0.67-2.45)	.45	N/A	N/A
	Very low or low	13.52 (12.02-15.17)	2.1(1.47-3.94)	1.82 (0.93-3.56)	.08	N/A	N/A
**Area of residence^g^**							
	Urban	68.04 (65.86-70.15)	3.05 (2.46-3.76)	Referent		N/A	N/A
	Regional or remote	30.34 (28.28-32.49)	2.21 (1.56-3.10)	0.72 (0.48-1.09)	.12	N/A	N/A
**High alcohol consumption^h^**							
	No	N/A	2.65(2.18-3.23)	Referent		N/A	N/A
	Yes	N/A	3.22(2.18-4.74)	1.22 (0.78-1.92)	.38	N/A	N/A
**Injected drugs in last year**							
	No	N/A	2.71 (2.26-3.27)	Referent		N/A	N/A
	Yes	N/A	5.31(2.59-10.57)	2.00 (0.93-4.33)	.08	N/A	N/A
**Smoking status**							
	Never smoked or former smoker	N/A	2.38 (1.92-2.95)	Referent		N/A	N/A
	Current smoker	N/A	4.37 (3.14-6.05)	1.87 (1.25-2.81)	.002	N/A	N/A
**STI^i^ testing in last year**							
	No test	N/A	1.43 (1.09-1.88)	Referent		Referent	
	STI test	N/A	10.98 (8.42-14.21)	10.92 (8.37-14.12)	<.001	2.02 (1.12-3.38)	.008
	STI diagnosis	N/A	31.09(17.57-48.86)	31.30 (14.07- 69.69)	<.001	3.15 (1.25-7.97)	.02
**Condom use with most recent partner**							
	Used condoms	N/A	5.25(3.92-6.99)	3.26 (2.11-5.04)	<.001	N/A	N/A
	Did not use	N/A	1.65 (1.23-2.21)	Referent	N/A	N/A	N/A
**Number of sexual partners in last year**							
	1	N/A	0.61 (0.36-1.1)	Referent		Referent	
	2-3	N/A	11.85 (9.12-15.24)	24.40 (13.82- 43.07)	<.001	17.86 (9.20-34.68)	<.001
	>3	N/A	22.85 (18.3-28.14)	69.64 (36.75- 131.97)	<.001	35.77 (18.04-70.94)	<.001

^a^n=8567 (2735), weighted (unweighted) denominators.

^b^Individuals with missing data are not shown; this was <5% for all variables, except for income and condom use with last sexual partner (not available for 42%).

^c^All data have been weighted to match the Australian population.

^d^Adjusted for age group, sexual identity, STI testing in the last year, and numbers of sexual partners in last year.

^e^N/A: not applicable.

^f^Too few responses for analysis (n<15).

^g^Accessibility/Remoteness Index of Australia (ARIA).

^h^≥28 standard drinks per week.

^i^STI: sexually transmissible infection.

In multivariate analyses, among sexually active men, several factors were associated with having had sex with someone met on a website or a smartphone app (Table 3): identifying as homosexual or bisexual compared with heterosexual (adjusted odds ratio, AOR: 15.37, 95% CI 8.34-28.35), having 2-3 sexual partners (AOR: 9.20, 95% CI 9.20-34.68) or >3 sexual partners in the last year (AOR: 35.77, 95% CI 18.04-70.94) compared with 1 partner, having STI in the previous year (AOR: 3.15, 95% CI 1.25-7.97), and having had an STI test in the previous year (AOR: 2.02, 95% CI 1.21-3.38) compared to not having an STI test.

#### Women

Among sexually active women, most were heterosexual (96.09%, 7838/8158), spoke English at home (96.25%,7852/8158), and lived in urban areas (67.78%, 5530/8158). Furthermore, 28.86% (2355/8158) were aged 16-29 years, 23.45% (1913/8158) were aged 30-39 years, 20.74% (1692/8158) were aged 40-49 years, 17.12% (1397/8158) were aged 50-59 years, and 9.83% (802/8158) were aged 60-69 years. Most sexually active women reported very high (23.16%, 1890/8158]) or high (22.36%, 1824/8158) annual household income compared with middle (17.68%, 1442/8158) or low and very low (16.73%, 1265/8158) annual household income.

Among sexually active women, those aged 16-29 years (2.38%, 55/2355) were most likely to report having had sex with someone met on a website or a smartphone app, and the least likely to report were those aged 60-69 years. Having had sex with someone met on a website or a smartphone app was higher among lesbian and bisexual women than among heterosexual women (5.51% [111/7838] vs 1.42% [18/319]). Women with low and very low annual household income (3.25%, 44/1365) more frequently reported having had sex with someone met using these technologies, compared with women with middle (0.58%, 8/1442), high (0.41% ,7/1824), and very high incomes (0.51%, 10/1890). Those with either an STI test (4.25%, 56/1330) or an STI diagnosis (9.99%, 13/130) in the last year were more likely to report having had sex with someone met online than those who had not had an STI test in the last year (0.89%, 59/6667). Women with 2-3 (13.37%, 59/438) or >3 (25.30%, 37/147) sexual partners in the last year were substantially more likely to report having had sex with someone met online than women with 1 sexual partner (0.44%,33/7574).

Among sexually active women, several factors were associated with having had sex with someone met online (Table 4): having 2-3 sexual partners in the last year (AOR: 32.01, 95% CI 13.17-77.78) or >3 sexual partners in the last year (AOR: 71:03, 95 % CI: 27.48-183.57), reporting a very low and low annual household income compared with very high annual household income (AOR: 3.40, 95% CI 1.12-10.35), and identifying as lesbian or bisexual (AOR: 2.27, 95% CI 1.04-4.49).

**Table 4:** Sociodemographic and behavioral correlates of having sex with someone met on a website or a smartphone app among sexually active women.

Characteristics^a,b^		%^c^ (95% CI) in subgroup		%^c^ (95% CI) outcome		Odds ratio (95% CI)		*P* value		Adjusted odds ratio (95% CI)^d^		*P* value	
**Age group (years)**													
	16-29		28.86 (26.36-31.51)		2.38 (1.62-3.38)		2.34 (1.62-3.38)		.29		0.43 (0.21 -0.86)		.02
	30-39		23.45 (21.39-25.63)		1.69 (1.04-2.72)		Referent		N/A^e^		Referent		N/A
	40-49		20.74 (18.91-22.70)		1.28 (0.68-2.38)		0.75 (0.34 - 1.68)		.49		1.16 (0.45 - 3.01)		.75
	50-59		17.12 (15.55-18.82)		1.07 (0.38-2.98)		0.63 (0.20-1.99)		.43		1.22 (0.36 -4.18)		.75
	60-69		9.83 (8.65-11.14)		0.60 (0.13-2.77)		0.35 (0.07- 1.79)		.21		0.66 (0.11 -3.98)		.65
**Sexual identity**													
	Heterosexual		96.09 (95.35-96.72)		1.42 (1.08 - 1.91)		Referent		N/A		Referent		N/A
	Lesbian, bisexual, or other		3.91 (3.28-4.65)		5.51 (3.12- 9.54)		4.05 (2.09 - 7.84)		<.001		2.27 (1.04-4.94)		.04
**Language spoken at home**													
	English only		96.25 (2.78-5.04)		0.84 (0.20 - 3.88)		N/A		N/A		N/A		N/A
	Other		3.75 (94.96-97.22)		—^f^		N/A		N/A		N/A		N/A
**Annual household income**													
	Very high		23.16 (21.15-25.31)		0.51 (0.25-1.06)		Referent		N/A		Referent		N/A
	High		22.36 (20.33-24.54)		0.41 (0.17-1.00)		0.79 (0.25 - 2.53)		.69		0.86 (0.28 - 2.67)		.80
	Middle		17.68 (15.83-19.69)		0.58(0.25-1.31)		1.12 (0.37 - 3.39)		.84		1.00 (0.31 - 3.23)		.99
	Very low or low		16.73 (14.95-18.68)		3.25 (2.05)		6.49 (2.71-15.55)		<.001		3.40 (1.12 - 10.35)		.03
**Area of residence^g^**													
	Urban		67.78 (65.44-70.03)		1.74 (1.26 - 2.40)		Referent		N/A		N/A		N/A
	Regional or remote		30.54 (28.34-32.82)		1.19 (0.79 - 1.78)		0.68 (0.40 - 1.14)		.12		N/A		N/A
**High alcohol consumption^h^**													
	No		N/A		1.44 (1.02 - 2.05)		Referent		N/A		N/A		N/A
	Yes		N/A		1.82 (1.24 - 2.67)		1.27 (0.75 - 2.15)		.38		N/A		N/A
**Injected drugs in last year**													
	No		N/A		1.57 (1.22 - 2.07)		Referent		N/A		N/A		N/A
	Yes		N/A		2.09 (0.64 - 6.76)		1.34 (0.39 - 4.62)		.65		N/A		N/A
**Smoking status**													
	Never smoked or former smoker		N/A		1.56 (1.18 - 2.12)		Referent		N/A		N/A		N/A
	Current smoker		N/A		1.69(1.03 - 2.81)		1.08 (0.60 - 1.97)		.81		N/A		N/A
**STI^i^ testing in last year**													
	No test		N/A		0.89 (0.59 - 1.36)		Referent		N/A		N/A		N/A
	STI test		N/A		4.25 (2.97 - 6.16)		4.94 (2.79 - 8.77)		<.001		N/A		N/A
	STI diagnosis		N/A		9.99 (4.93 - 19.20)		12.38 (5.17 - 29.62)		<.001		N/A		N/A
**Condom use with most recent partner**													
	Used condoms		N/A		3.41 (2.24 -5.15)		1.06 (0.57-1.96)		.86		N/A		N/A
	Did not use		N/A		3.60 (2.34 -5.48)		Referent		N/A		N/A		N/A
**Number of sexual partners in last year**													
	1		N/A		0.44 (0.22-0.88)		Referent		N/A		Referent		N/A
	2-3		N/A		13.37 (9.93-17.77)		35.13 (16.12-76.59)		<.001		32.01 (13.17-77.78)		<.001
	>3		N/A		25.30 (17.20 -35.58)		77.08 (32.75-181.43)		<.001		71.03 (27.48 -183.57)		<.001

^a^n=8158 (2580), weighted (unweighted) denominators

^b^Individuals with missing data are not shown; this was <5% for all variables, except for income and condom use with most recent partner (not available for 42%).

^c^All data have been weighted to match the Australian population.

^d^Adjusted for age group, income, and numbers of sexual partners in the last year.

^e^N/A: not applicable.

^f^Too few responses for analysis (n<15).

^g^Accessibility/Remoteness Index of Australia.

^h^≥14 standard drinks per week (for women).

^i^STI: sexually transmissible infection.

## Discussion

### Principal Findings

Overall, our findings indicate that in 2012-2013, approximately 1 in 10 Australian adults aged from 16 to 69 years had ever looked for potential partners using websites or smartphone apps, of whom approximately half had done so in the in the last year. Among people who searched in the last year, over half had physically met with someone, and approximately two-thirds of these people had had sex with someone they met online in the last year, equating to 1.95% of the population.

These nationally representative estimates of looking for and having had sex with someone met on website and smartphone apps are lower than those of surveys focusing on specific subpopulations and using convenience-sampling frames, which have reported 6%-40% of the population meeting sexual partners using websites [2,5,11,12] and 18%-76% using dating applications and websites [3,13-18]. The difference is almost certainly related to different populations sampled and may also be related to the fact that ASHR2 asked specifically about having sex with someone, rather than only looking for partners or meeting in person. Age was strongly correlated with having had sex with someone met on a website or a smartphone app, especially since younger people tend to have higher levels of mobile phone and Internet access, but this study included a broader range of ages than many others. Just over a third (36.07%) of people who used internet dating and hookup applications in the last year reported having had sex with someone they met on a website or using a smartphone app in the last year. Certain populations were less likely to report having had sex with someone met using these technologies. For example, 30.89% of heterosexual respondents who reported that they had used the Internet or a mobile phone app to search for a potential partner in the last year reported having had sex with someone they met online, compared with 62.41% of gay, lesbian, and bisexual survey respondents (see Table 2). This suggests that other studies reporting on the use of internet dating and hookup apps as a proximal marker of having had sex with someone met online are likely to overestimate the practice’s prevalence among lower-risk segments of the population.

In this survey, 15.58% of gay, lesbian, and bisexual respondents reported meeting a sexual partner in the last year. Higher uptake of finding partners using websites among nonheterosexual respondents was also observed in a British population survey [4]. Yet, in our study, after adjusting for age and other characteristics, the strongest correlate of having had sex with someone met using a website or smartphone app was higher numbers of sexual partners in the last year, suggesting that these technologies are favored by the most sexually active respondents. This finding is consistent with other studies reporting that people who look for partners with these tools have increased sexual activity compared with nonusers, including younger age at first sex [2,5] and higher numbers of sexual partners [2,4,5,12,15,16]. Due to the cross-sectional nature of the survey, determining causality is not possible, so findings could mean platforms provide an efficient means for more sexually active individuals to connect with new partners, or alternatively, people who were already more sexually active were attracted to these sites and other ways to meet sexual partners.

In general, people who met partners using websites and apps and had sex with them were more likely to engage in higher-risk practices than those who did not—except for condom use at the last sexual event, which was higher among people who met partners online. There was attenuation of the condom use variable in the multivariate analysis, meaning that the association was not significant in the adjusted analysis after controlling for the numbers of sexual partners and other demographic factors. Higher levels of condom use at the last event could reflect condom use with newer and less established partners, with whom STI prevention is prioritized. This explanation seems highly plausible because online tools are often used to facilitate new sexual partnerships and those who report using websites and apps to find sexual partners also report higher numbers of recent sexual partners. However, the finding contrasts with many other studies that tend to find meeting or seeking partners online is linked to condomless sexual intercourse [4,5,14,15].

In relation to STI history, both STI testing and diagnoses were higher among people who reported meeting partners using websites and smartphone apps and having sex with them. Again, adjusted analysis showed attenuation of STI history for women after adjusting for the number of sexual partners. This implies that women who reported either an STI test or diagnosis in the last year were also more likely to have multiple sexual partners in the last year. The relationship between STI history and meeting partners using websites and apps remained significant for men, even after adjusting for partner numbers and other demographic factors. Interestingly, this pattern has also been seen in the other two population studies with a significant relationship between STIs and searching for or meeting partners on websites and smartphone apps among men, but not among women [4,5]. Findings from other studies have been mixed as to whether STI diagnoses are related to either finding or searching for sexual partners on internet sites and geosocial apps [1-4,16,19]. A number of studies have reported on HIV testing history among gay men and tend to find that men who use apps are more likely to have been tested for HIV [14,19]. Studies with gay men in Australia suggest that men who use a combination of mobile phone apps, internet websites, and offline places to meet partners appear to be at increased risk of STIs or HIV compared with men who use a narrower range of online and offline methods [20].

In women, low annual household income was associated with meeting partners on websites and apps. Socioeconomic deprivation has been linked with poor health outcomes, including STI acquisition [21-23], and other reports from ASHR2 found lower income related to multiple sexual partners [24]. Aside from age and income level for women, other sociodemographic factors, smoking, high alcohol consumption, and injecting drug use were not associated with having sex with someone met online.

### Limitations

To our knowledge, this is the first study to report the prevalence of having had sex with someone met on a website or a mobile phone app in the past year from a representative survey of the general adult population. One of this study’s strengths is the capacity to assess the proportion of people who used internet dating and apps, met in person, and had sex with someone met online within the same population. Nonetheless, our study also has several limitations to consider when interpreting findings. First, the study was conducted in 2012-2013, and since that time, the technology landscape and behaviors related to the uptake of technology have changed. Very likely, the uptake of dating and hookup apps has substantially increased since the survey was conducted. Second, the sample of homosexual and bisexual men was not sufficiently large to enable analyses focused on this group. Third, all outcomes including STI outcomes were based on self-report, which is susceptible to recall and other reporting biases. Several similar studies have used biological measurement to ascertain STI prevalence, a more robust measure [2,4,5]. However, when asked, 88.55% of participants reported answering all survey questions truthfully, and a further 9.89% reported that they had answered 90%-99% of the survey honestly. This equates to 98.44% of participants answering at least 90% of questions truthfully [7]. Furthermore, the clear majority (89.97%) of participants reported that they were either not embarrassed or only slightly embarrassed by the questions. Evidence of the relatively low embarrassment and discomfort with questions related to sexual practices is seen in the number of people declining to answer particular questions. The question with the highest rate of refusal involved annual income compared with much lower refusal rates for questions about sexual practices .

### Implications

Understanding the number and characteristics of people most likely to use these technologies to meet new sexual partners assists organizations responsible for HIV and STI prevention programs in identifying places and populations wherein they can focus their health promotion and testing initiatives. Our study has also demonstrated that although the prevalence of having had sex with someone met on a website or a smartphone app was 3.03% in people aged 20-29 years, it remained at 2.25% in people aged 30-49 years, suggesting the need for promotional material to cover a broad range of ages, not just younger adults. Although STIs are most prevalent in people aged 16-29 years, recent studies have suggested an increased rate in people over 30 years [25]. Furthermore, despite the finding that people who met partners on dating websites and apps were more likely to have had an STI test in the last year, a substantial proportion of respondents who reported having sex with someone they met online (likely to be a new sexual partner) reported not having an STI test in the past year, suggesting an opportunity to raise awareness about STI testing further and to use targeted advertisements to direct people to easy access points such as new websites where pathology request forms can be downloaded without attending a clinic [26]. Some have also suggested that these platforms have the capacity to enable partner notification and data collection in relation to sexual health [27]. Notably, however, the owners of dating websites and apps have historically been concerned about associating their platforms with STIs and therefore are reluctant to promote public health initiatives [27].

### Conclusions

Internet and smartphone technologies are a relatively common way of meeting new sexual partners among highly sexually active survey respondents, homosexual and bisexual men, and younger adults, suggesting that the use of in-app health promotion is a feasible approach to targeting these populations [28,29]. Future research could explore the potential of health promotion in dating websites and geosocial applications. The use of smartphone technologies to search for potential sexual partners may become a normative dating practice among Australian adults in their twenties and thirties, so repeat surveys would be important to document this prevalence over time.

## Appendix

Multimedia Appendix 1 Prevalence of looking for potential partners on websites or smartphone apps and prevalence of having sex with these partners among males.

Multimedia Appendix 2 Prevalence of looking for potential partners on websites or smartphone apps and prevalence of having sex with these partners among females.

## Abbreviations

ASHR2: Second Australian Study of Health and Relationships
AOR: adjusted odds ratio
STI: sexually transmissible infection
