# Supplementary material for: Meeting Sexual Partners Through Internet Sites and Smartphone Apps in Australia: National Representative Study
Source: J Med Internet Res. 2018 Dec 18;20(12):e10683. doi: 10.2196/10683 (PMC6315227; doi:10.2196/10683)
Supplement: Multimedia Appendix 1 [file jmir_v20i12e10683_app1.pdf]

Multimedia Appendix 1. Prevalence of looking for potential partners on websites or smartphone apps and prevalence of having sex with these partners among males.

Characteristic		Searched for potential partner- ever	Searched for potential partner- last year	Met in person-last year	Had sex- last year
		%[a] (95% CI)	%[a] (95% CI)	%[a] (95% CI)	%[a] (95% CI)
All participants [b]					
**Age group (years)**					
	16-29	17.70 (14.68-21.18)	11.11 (8.98-13.67)	6.31 (4.82-8.23)	4.12 (3.07-5.52)
	30-39	18.40 (15.00-22.37)	8.95 (6.73-11.79)	4.68 (3.16-6.87)	2.93 (1.98-3.32)
	40-49	12.46 (10.15-15.20)	6.06 (4.74-7.73)	3.03 (2.24-4.08)	2.39 (1.71 -3.35)
	50-59	10.62 (8.52-13.17)	4.66 (3.57-6.07)	2.17 (1.48-3.15)	1.62 (1.06-2.47)
	60-69	5.39 (4.04-7.15)	1.93 (1.39-2.67)	1.04 (0.67-1.62)	0.69 (0.40-1.18)
**Sexual identity**					
	Heterosexual	12.05 (10.79-13.43)	5.96 (5.17-6.87)	2.80 (2.28-3.44)	1.67 (1.33 -2.09)
	Homosexual or lesbian or bisexual	57.99 (51.03-64.65)	38.89 (32.47-45.73)	31.59 (25.59-38.26)	28.82 (23.02-35.40)
**Language spoken at home**					
	English only	13.41 (12.14-13.78)	6.73 (5.95 -7.61)	3.64 (3.11-4.27)	2.62 (2.20-3.11)
	Other	15.29 (9.52 -23.66)	11.47 (6.62 -18.5)	4.94(1.92-12.16)	1.18 (0.37-3.75)
**Annual household income**					
	Very low or low	12.67 (9.72-16.34)	7.97 (5.54-11.33)	4.28 (2.61-6.95)	2.41 (1.47-3.94)
	Middle	12.54 (9.50-16.38)	6.96 (5.08 -9.45)	2.50 (1.65-3.77)	1.79 (1.10-2.89)
	High	11.64 (8.87-15.14)	3.25 (2.19-4.79)	1.22 (0.74-2.01)	0.67 (0.34-1.32)
	Very high	9.40 (7.26-12.10)	3.51 (2.37-5.19)	2.00 (1.16-3.43)	1.42 (0.94-2.12)
**Area of residence[c]**					
	Urban	14.21 (12.61-15,98)	7.06 (6.05-8.21)	4.14 (3.39-5.05)	2.77 (2.26-3.39)
	Regional or remote	11.94 (10.05-14.14)	6.93 (5.58-8.58)	2.82 (2.14-3.72)	2.01 (1.44-2.80)
**High alcohol consumption**					
	No	13.52 (12.08-15.09)	6.93 (6.00-7.99)	3.54 (2.95-4.25)	2.41 (1.99-2.91)
	Yes	13.52 (11.05-16.45)	7.29 (5.71-9.26)	4.29 (3.01-6.07)	2.9 2(2.01-4.20)
**Injected drugs in last year**					
	No	13.39 (12.11-14.78)	6.88 (6.05-7.82)	3.66 (3.09-4.34)	2.48 (2.08 -2.96)

	Yes	18.00 (12.09-25.93)	11.41 (7.33-17.34)	5.50 (2.97-9.98)	4.40 (2.16-8.73)
**Smoking status**					
	Never smoked/ former	11.98 (10.64-13.5)	5.78 (4.96-6.73)	3.00 (2.46-3.64)	2.16 (1.75-2.65)
	Current smoker	19.87(16.68-23.49)	12.12 (9.81-14.88)	6.66 (4.93-8.93)	3.99 (2.91-5.46)
**STI[d] testing in last year**					
	No test	10.06 (8.72-11.58)	4.25 (3.48-5.18)	2.04 (1.57-2.65)	1.49 (1.11-1.89)
	STI test	30.86 (25.63-36.63)	20.87 (16.74-25.71)	14.97 (11.49-19.28)	11.04 (8.50-14.23)
	STI diagnosis	59.35 (40.22-76.01)	40.03 (23.85-58.71)	32.94 (18.95-50.80)	30.09 (17.57-48.85)
**Condom use with most recent partner**					
	Used condoms	16.61 (13.67-20.04)	11.4 (9.17 -14.11)	6.52 (5.02-8.43)	5.24 (3.92-6.99)
	Did not use	10.99(9.33-12.91)	3.81 (3.00 -4.84)	2.29 (1.68-3.12)	1.64 (1.22-2.20)
**Number of sexual partners in last year**					
	1	10.08 (8.79-11.54)	3.79 (3.06-4.69)	1.36 (0.93-1.98)	0.58 (0.36-0.93)
	2-3	35.37(31.07-39.92)	26.90 (22.96 - 31.25)	16.35 (13.13-20.19)	11.85 ((9.12-15.24)
	>3	41.21 (35.56 - 47.10)	34.01 (28.7 - 39.76)	26.82 (21.98-32.33	23.32 (18.80 - 28.55)

[a]All proportions have been weighted to match the Australian population.
[b] n=9761 (3890)., weighted (unweighted) denominators.
[c]Accessibility/Remoteness Index of Australia.
[d]STI: sexually transmissible infection.
