# Supplementary material for: Meeting Sexual Partners Through Internet Sites and Smartphone Apps in Australia: National Representative Study
Source: J Med Internet Res. 2018 Dec 18;20(12):e10683. doi: 10.2196/10683 (PMC6315227; doi:10.2196/10683)
Supplement: Multimedia Appendix 2 [file jmir_v20i12e10683_app2.pdf]

Multimedia Appendix 2. Prevalence of looking for potential partners on websites or smartphone apps and prevalence of having sex with these partners among females.

Characteristic		Searched for potential partner- ever	Searched for potential partner- last year	Met in person - last year	Had sex- last year
		%[a] (95% CI)	%[a] (95% CI)	%[a] (95% CI)	%[a] (95% CI)
All participants[b]					
**Age group (years)**					
	16-29	11.02 (8.56-14.09)	5.81 (4.23-7.93)	3.25 (2.41-4.37)	2.06 (1.43-2.96)
	30-39	13.50 (10.90-16.61)	3.75 (2.79-5.02)	2.51 (1.72-3.65)	1.61 (0.99-2.60)
	40-49	11.16 (9.01-13.74)	3.39 (2.41-4.76)	1.74 (1.11-2.73)	1.18 (0.64-2.15)
	50-59	9.37 (7.46-11.71)	2.30 (1.42-3.71)	1.81 (0.99-3.28)	0.84 (0.30-2.36)
	60-69	6.37 (4.72-8.55)	1.75 (1.12-2.73)	0.92 (0.44-1.92)	0.38 (0.08-1.76)
**Sexual identity**					
	Heterosexual	9.88 (8.76-11.13)	3.33 (2.74-4.03)	1.99(1.61-2.45)	1.21 (0.91-.1.61)
	Homosexual or lesbian or bisexual	29.93 (24.11-36.48)	13.43 (9.61-8.46)	8.14 (5.30-12.32)	4.77 (2.71-8.25)
**Language spoken at home**					
	English only	10.73 (9.61-11.98)	3.67 (3.10-4.35)	2.27 (1.87-2.75)	1.37 (1.06-1.78)
	Other	8.48 (3.71-18.24)	4.89 (1.44-15.32)	1.42 (0.53-3.37)	0.72 (0.15-3.30)
**Annual household income**					
	Very low or low	11.28 (9.18-11.78)	5.5 (4.13-7.29)	3.79 (2.62-5.45)	2.58 (1.63-4.05)
	Middle	8.52 (6.41-11.24)	2.46 (1.66-3.63)	1.37 (0.82-2.27)	0.52 (0.23-1.20)
	High	7.43 (5.35-10.22)	1.62 (0.69-3.74)	0.66 (0.33-1.31)	0.44 (0.19-1.01)
	Very high	10.24 (7.46-13.91)	1.58 (0.89-2.78)	1.00 (0.59-1.72)	0.49 (0.19-1.02)
**Area of residence[c]**					
	Urban	11.62 (10.18-13.22)	4.11 (3.33-5.06)	2.59 (2.07-3.23)	1.48 (1.08-2.03)
	Regional or remote	8.51 (6.94-10.40)	2.88 (2.15-3.86)	1.42 (1.00-2.00)	1.02 (0.68-1.51)
**High alcohol consumption**					
	No	11.23 (9.83-12.79)	3.81 (3.07-4.72)	2.15 (1.68-2.75)	1.22 (0.87-1.73)
	Yes	9.57 (7.84-11.62)	3.55 (2.67-4.72)	2.36 (1.74-3.18)	1.58 (1.07-2.31)
**Injected drugs in last year**					
	No	10.47 (9.35-11.71)	3.63 (3.04-4.34)	2.20 (1.81-2.67)	1.33 (1.02-1.73)
	Yes	20.89(13.34-31.17)	8.84 (5.14-14.79)	3.89 (1.78-8.28)	2.28 (0.82-6.19)
**Smoking status**					

	Never smoked/ former	10.18 (8.90-11.62)	3.45 (2.77-4.28)	2.20 (1.76-2.75)	1.41 (1.05-1.88)
	Current smoker	12.76 (10.35-15.64)	5.12 (3.90-6.69)	2.57 (1.73-3.79)	1.44 (0.87-2.38)
**STI [d] testing in last year**					
	No test	8.07 (6.87-9.45)	2.24 (1.66-3.01)	1.32 (0.94-1.85)	0.89 (0.58-1.35)
	STI test	18.45 (14.06-23.82)	7.84 (5.45-11.16)	5.36 (3.86-7.40)	4.34 (3.02-6.21)
	STI diagnosis	25.86 (16.34-38.37)	19.04 (11.25-30.39)	15.26 (8.5-25.88)	9.84 (4.86-18.89)
**Condom use with most recent partner**					
	Used condoms	12.2 (0.92-16.05)	6.70 (4.83-9.22)	4.83 (3.35-6.93)	3.60 (2.34-5.48)
	Did not use	18.31 (13.54-24.29)	8.23 (5.47-12.44)	4.99 (3.45-7.18)	3.49 (2.32-5.24)
**Number of sexual partners in last year**					
	1	9.27 (8.15-10.53)	2.36 (1.83-3.05)	1.05(0.76-1.44)	0.37 (0.18-0.74)
	2-3	31.47 (26.21-27.25)	22.96 (18.41-28.25)	17.86 (13.81-22.77)	13.39 (9.94-17.79)
	>3	33.93 (24.66-44.61)	31.14 (22.29-41.60)	28.82 (20.20-39.32)	26.30 (18.2-36.69)

[a]All proportions have been weighted to match the Australian population.
[b] n=9637 (4294),weighted (unweighted) denominators.
[c]Accessibility/Remoteness Index of Australia.
[d]STI: sexually transmissible infection.
